# Blastocystis and Schistosomiasis Coinfection in a Patient with Chronic Kidney Disease

**DOI:** 10.1155/2014/676395

**Published:** 2014-10-19

**Authors:** Colin R. Young, Fred E. Yeo

**Affiliations:** ^1^Walter Reed National Military Medical Center, Medical Corps, United States Navy, 8901 Rockville Pike, Bethesda, MD 20889, USA; ^2^Naval Health Clinic New England, Clinic Groton Branch, 1 Wahoo Drive, Groton, CT 06349, USA

## Abstract

Chronic kidney disease (CKD) and end-stage renal disease (ESRD) represent a spectrum of impaired immunity with effects on cellular immunity, soluble immune factors, and inflammation. As a result, infections due to impaired immune system responses are responsible for significant morbidity in patients with kidney disease. Because of immune dysfunction in CKD, these patients have reduced probability to clear infections and are susceptible to pathogenic effects of common organisms. We present a case of a patient with CKD coinfected with *Schistosoma mansoni* and *Blastocystis* spp. This appears to be the first reported association of *Schistosoma mansoni* and *Blastocystis* spp. in a patient with CKD.

## 1. Introduction

Infections are a leading cause of morbidity and mortality in patients with chronic kidney disease (CKD), and much of this morbidity is attributed to immune dysfunction associated with progressive chronic renal impairment [1]. While most attention is given to patients with end-stage renal disease (ESRD), milder degrees of CKD (stages 3, 4, and 5) have become increasingly associated with altered immunity and infection related morbidity [2]. Accumulation of uremic toxins with altered T-cell and B-cell functions is thought to play a central role in the immune alterations that take place with progressive renal disease. Altered immunity in progressive CKD and ESRD is associated with bacteremia, sepsis, and infections with severity not typically encountered in the general population [3]. This case involves coinfection with* Schistosoma mansoni* and* Blastocystis* spp. in a patient with chronic kidney disease (CKD). This appears to be the first report of such an association.

## 2. Case Report

A 37-year-old male US Navy enlisted laboratory technician presented for his annual military physical health assessment, where his serum creatinine was noted to be 1.4 mg/dL and his blood urea nitrogen level was 24 mg/dL. He was subsequently referred for further evaluation. The patient felt well and denied any renal or urologic symptoms, use of nonsteroidal anti-inflammatory medications, recurrent kidney stones, or recent intravenous contrast imaging studies. On review of systems, the patient endorsed chronic intermittent abdominal pain occasionally associated with diarrhea and chronic left hip pain attributed to a sports injury. The patient was born and raised in Liberia until the age of 16 when his family immigrated to the United States. He traveled from the United States to Liberia at least twice yearly for two-week periods to visit relatives who reside in arid rural communities. The patient's medical history was notable for diet-controlled hyperlipidemia, a left hip labral tear with operative repair one year ago, and treatment with isoniazid for 9 months for latent tuberculosis 10 years ago. The patient had numerous emergency room visits for exacerbations of abdominal pain associated with diarrhea. The patient described several specialty evaluations for his abdominal symptoms and had been given the diagnosis of medically refractory irritable bowel syndrome.

**Figure 1:**
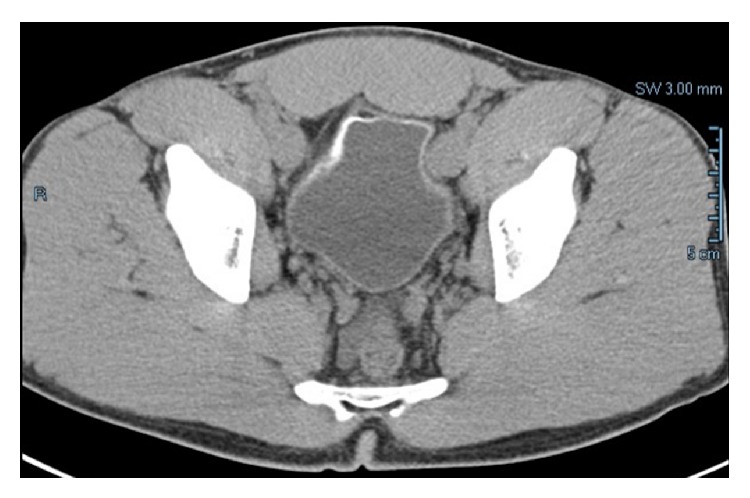
Axial section showing circumferentially calcified bladder.

**Figure 2:**
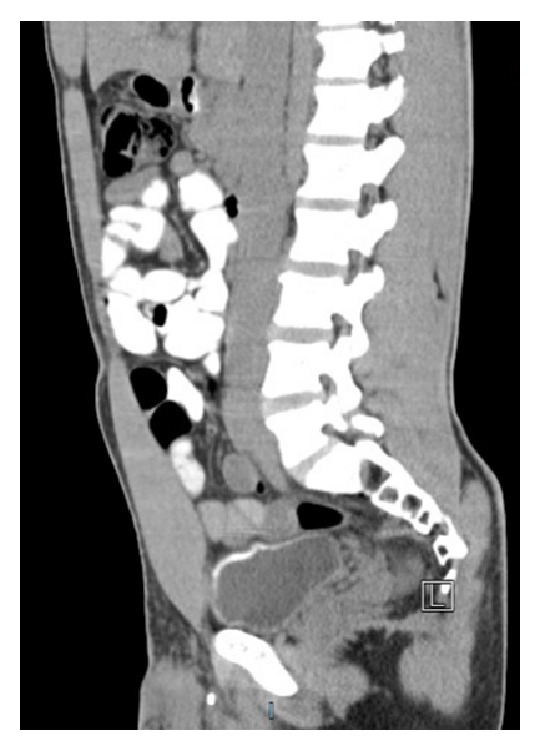
Sagittal section showing circumferential calcified bladder.

An acute care visit 5 years prior to this presentation for abdominal pain and lethargy was notable for a serum creatinine of 2.2 mg/dL, microscopic hematuria, and proteinuria. Computed tomography scanning at that time revealed a moderate amount of free fluid in the pelvis and circumferential bladder calcification (Figures 1 and 2). He received a diagnosis of dehydration and was treated with intravenous fluids. He was referred to urology for cystoscopy, which was reportedly unremarkable; however no bladder biopsies were performed.

Physical examination revealed a temperature of 37 degrees Celsius, blood pressure of 110/80 mmHg, and heart rate of 70 beats per minute. The patient appeared well. Other than mild left hip point-tenderness, the physical examination was entirely normal and specifically did not reveal lymphadenopathy, cardiac murmurs, hepatosplenomegaly, rashes, or edema.

Serum electrolytes, liver associated enzymes, and serum intact parathyroid hormone levels were normal. Complete blood count was normal except for a hemoglobin level of 13.5 g/dL. Dipstick urinalysis did not reveal any abnormalities. Urine sediment analysis revealed numerous parasitic cyst forms and granular casts. Renal ultrasound imaging revealed small mildly echogenic kidneys consistent with chronic kidney disease. Tests for hepatitis B, hepatitis C, and human immunodeficiency virus were negative. Testing for urinary schistosomiasis revealed organisms identified as* Schistosoma mansoni*. Specific blood testing for schistosomal antibodies revealed an antischistosomal immunoglobulin G antibody level of 1.76 (normal 0-1.00). Stool studies for ova and parasites revealed* Blastocystis* spp. (8 per high power field).

## 3. Discussion

Schistosomiasis is associated with several patterns of renal disease including schistosomal glomerulopathy [4], chronic pyelonephritis [5], obstructive renal failure [6], and chronic granulomatous disease. Schistosomiasis has been reported as a coinfection with* Salmonella* [7] and other organisms in cases of renal disease; however coinfection with* Blastocystis* spp. has not been reported in a patient with CKD, to our knowledge.* Blastocystis* spp. has been historically thought of as a commensal parasite with little potential for pathogenicity [8]. Over the past several decades, the clinical significance of this parasite has been reexamined due to increased reports of symptomatic infection without other attributable etiologic agents, associations with other comorbid illness, and more frequent occurrences of invasive species in susceptible populations [9]. However, there is little published literature describing infection with* Blastocystis* spp. in CKD. There have been reports of* Blastocystis* spp. in patients on dialysis; however it remains to be seen if this is a true association or a coincidental finding [10].

In individuals with an intact and normally functioning immune system, the majority of cases of genitourinary schistosomiasis or intestinal* Blastocystis* spp. infections result in subclinical presentations and are thought to spontaneously regress. Progressive kidney disease, however, is associated with altered and diminished T-cell and B-cell immune responses and impaired immune cascades [3], and organisms generally thought of as commensals can result in significant morbidity. Accumulation of uremic toxins and the effects of these toxins are thought to be the etiology of immune dysfunction in CKD and ESRD. Unfortunately, a specific uremic toxin has not been identified, despite several promising candidates, though likely involves a combination of so-called “middle molecules,” low-molecular-weight solutes, and imbalance between pro- and anti-inflammatory soluble mediators [2]. Traditional views of immunity in CKD and ESRD suggest that worsening immune dysfunction is directly correlated with the degree of renal dysfunction. Recently, however, this thinking has been challenged, with increasing reports of opportunistic infections in patients with milder degrees of renal impairment [11]. Specific immune alterations have been demonstrated in CKD including progressive B-cell lymphocytopenia, increased apoptosis associated with reduced IL-7 levels, and shifts in T-cell lymphocyte subset populations [12]. It is likely that immune dysfunction and accumulation of uremic toxins are intimately involved with renal immunosuppression and clinically manifest earlier in chronic kidney disease than traditionally appreciated.

This case illustrates a coinfection with* Schistosoma mansoni* and* Blastocystis* spp. and incidentally noted CKD. It is not entirely clear if the CKD preceded the schistosomiasis and blastocystis infections or if the CKD occurred as a result of the schistosomal infection. Given that the patient was born and raised in Liberia, a known endemic reservoir for* Schistosoma mansoni*, and had no other risk factors for CKD, we suspect that the patient's chronic untreated infection with schistosomiasis and genitourinary involvement resulted in CKD. We postulate that he acquired* Blastocystis* spp. and that infection flourished secondary to reduced immunity from his CKD or through a pathologic symbiotic relationship with* Schistosoma mansoni*. This chronic blastocystis infection likely accounts for his diagnosis of “refractory irritable bowel syndrome,” since his abdominal symptoms resolved promptly after treatment, initially with metronidazole for his blastocystis, and then subsequently with praziquantel for schistosomiasis, and he has been symptom free for more than 1 year with eradication verified by stool sampling. We acknowledge the remote possibility that all three conditions may have occurred independently and are unrelated, though the time course, data, imaging, and response to treatment presented in this report provide a compelling argument against this.

This case highlights several important clinical concepts. Chronic kidney disease of even mild degrees represents a state of impaired immunity and the clinician should maintain a heightened sense of awareness for subclinical presentations of opportunistic infections, especially in people who reside or originate from endemic regions. Coinfections should serve as a clue to altered immunity and investigation should be undertaken to determine the cause and nature of the immune dysfunction. Clinicians should be aware of the fact that even mild CKD represents a state of altered and impaired immunity. Despite the literature disagreement, treatment of infections in patients with CKD should be considered, as infections are a significant cause of morbidity and mortality in patients with CKD and ESRD, and eradication is necessary prior to kidney transplant considerations. Lastly, there may be a commensal relationship between* Schistosoma mansoni* and* Blastocystis* spp. that may be amplified in the setting of CKD, the nature of which is incompletely understood.
